# Imbalance polarization of M1/M2 macrophages in miscarried uterus

**DOI:** 10.1371/journal.pone.0304590

**Published:** 2024-07-25

**Authors:** Jun Feng, Ping Gao, Ting Wu, Wenjie Hou, Yueming Zhang, Lili Li

**Affiliations:** 1 Department of Obstetrics and Gynecology, The Fourth Affiliated Hospital of Soochow University, Suzhou, Jiangsu, China; 2 National Key Laboratory of Immunity and Inflammation, Suzhou Institute of System Medicine, Chinese Academy of Medical Sciences & Peking Union Medical College, Suzhou, Jiangsu, China; 3 Department of Obstetrics and Gynecology, Lixin Hospital of Chinese Medicine, Bozhou, Anhui, China; Universite du Quebec a Trois-Rivieres, CANADA

## Abstract

**Background:**

Lipopolysaccharides (LPS) is well known to manifest a miscarriage-inducing effector during early pregnancy and activate macrophage to induce M1 macrophage polarization. However, the role of macrophage polarization in LPS-related miscarriage-inducing effect is not apparent.

**Methods:**

In this work, gene expression changes and the percentage of M1/M2 macrophages and monocytes in LPS-induced miscarried uterus were firstly analyzed by RNA sequencing (RNA-seq) and Flow Cytometry. To explore the origin that contributes to M1/M2 macrophage differentiation, the expression of monocyte chemotactic protein (MCP-1), CCL3, and CCL4, chemokines related to monocyte/macrophage migration, was tested by quantitative real time PCR (qRT-PCR).

**Results:**

We found that percentage of M1 macrophages rose, while the percentage of M2 macrophages declined down in the injected mice uterus. Meanwhile, the percentage of M1 and M2 macrophages showed no significant difference in the spleens of LPS injected mice compared to PBS injected control mice. Expression of *Mcp-1*, *Ccl3*, and *Ccl4* and numbers of monocytes were remarkably up-regulated in LPS-induced miscarried mice uterus.

**Conclusion:**

These results indicated that polarization and proportion changes of macrophage in the uterus may contribute to miscarriage. Our work provides new evidence correlating the aberrant regulation of M1/M2 macrophage polarization with deleterious miscarriage-inducing effects. This will help us understand the roles of critical immune cell differentiation in maintaining normal pregnancy.

## Introduction

Macrophage is a kind of leukocyte that significantly impacts pregnancy [1–3]. Macrophage takes up about 20% of the myeloid immune cells in human decidua, second only to NK cells [4, 5]. Although number of macrophages in mouse uterus is much lesser than that in humans and fluctuates with the pregnancy process, they have an essential impact on pregnancy [6, 7]. Two distinct states of polarized activation for macrophages have been defined: the classically activated macrophage phenotype and the alternatively activated macrophage phenotype [8–10]. Classically activated macrophages are effector cells in Th1 cellular immune response (M1 macrophage). The alternatively activated macrophages appear to be involved in the immunosuppression and tissue-repairing process, taking part in the Th2 cellular immune response (M2 macrophage) [11–14]. The existence of M1/M2 macrophages at the maternal-fetal interface is thought to impact pregnancy through regulating Th1/Th2 balance. The amount and percentage of M1/M2 macrophages are significant in keeping normal pregnancy [15–17].

Many research have found that the balance of M1/M2 macrophage proportion in the uterus significantly impacts early pregnancy [18, 19]. M1 macrophages induce pro-inflammatory response and host defense upon uteroplacental infection; M2 macrophages take up to 50% of total macrophages in early to mid-pregnancy uterus and are thought to exert immunosuppressive effect and mediate fetal-maternal immune tolerance [5, 20–22]. Successful pregnancy needs to keep a proper percentage of M1 and M2 macrophage in the uterus. Lots of reproduction-associated diseases, like eclampsia, recurrent miscarriage, and premature labor have been found to relate to M1 or M2 macrophages abnormal in the uterus in humans [3, 23–25]. So, regulating M1 and M2 macrophages is exceptionally vital to maintain normal pregnancy.

Lipopolysaccharides (LPS) also is known as endotoxins because of their bacteria- toxic effect [26–28]. LPS was found to have a miscarriage-inducing effect by Zahl and Bjerkness in 1943, and plenty of research has been conducted to investigate the mechanism of the miscarriage-inducing function of LPS [29]. Several explanations have been proposed: (1) LPS has toxic effect on placenta and results in fetal death [30, 31]. (2) LPS disturbs the uteroplacental circulation and causes thrombus formation, decidual obstructive necrosis, and placenta bleeding [32, 33]. (3) LPS triggers a systemic inflammatory reaction and leads fetal-maternal interface’s Th1/Th2 immune state to Th1 dominant. (4) LPS also can influence the phenotype and function of immune cells in the pregnancy uterus, such as Treg, DC, NK cells, and macrophages [34]. Despite these studies, the detailed process of how LPS regulates M1/M2 macrophage changes in a pregnant uterus is unclear.

In this work, we investigated the change of M1 and M2 macrophages in LPS-injected pregnant mice’s uteruses. Transcriptome sequencing results suggested that LPS regulated macrophage-associated genes and pathways in LPS-induced miscarried uterus. We then analyzed the M1/M2 macrophages and monocytes in the uterus at 3 h, 6 h, 12 h, and 24 h after LPS injection by Flow Cytometry experiments. The results showed that the percentage of M1 macrophage raised up and the percentage of M2 macrophage declined gradually after LPS administration. Gene expression analysis with q-PCR assay also showed that chemokines related to monocyte-macrophage recruitment (*Ccl3*, *Ccl4*, and *Mcp-1*) were also up-regulated in LPS-injected mice uterus and human miscarried placentas when compared with normal pregnant placentas. These results indicate that the LPS-induced miscarriage effect may correlate with the imbalance polarization of M1/M2 macrophages in uterus. This work highlights the critical role of M1/M2 macrophage balance in early pregnancy and helps us in understand human miscarriage.

## Materials and methods

### Experimental animals

Eight- to ten-week-old inbred C57BL/6 mice were purchased from Spf Animals Laboratories (SPF, Beijing, China). Mice housed in a temperature and humidity-controlled room with a constant photoperiod (12L: 12D) were fed *ad libitum* and had free access to tap water. Pregnancy was achieved by caging female mice with fertile males at a ratio of 2:1, and the day when a copulatory plug was observed was termed gestational day 1 (GD1).

### Transcriptome sequencing

Syngeneically mated Balb/c females were injected with LPS intraperitoneally on GD5 and sacrificed by CO_2_ asphyxiation on GD6. Miscarried uterus from LPS administration mice and non-miscarried uterus from PBS administration mice were collected and put into liquid nitrogen quickly. Uteri were obtained from three mice of each group, respectively, and then were sent to Beijing Genomics Institute company for transcriptome sequencing (Illumina Hiseq2000).

### Flow cytometry analysis

Pregnant mice were euthanized at various times (3 h, 6 h, 12 h, and 24 h) after LPS (Sigma Aldrich, 4 μg per mouse) or PBS control administration. Spleen were immediately collected and ground in a 35 mm plate containing 2 ml PBS and then sifted through a 37 μm cell strainer. Cells were depleted of RBCs using an ammonium chloride lysing solution (0.14 M NH_4_Cl, 10 nM KHCO_3,_ and 1 nM EDTA). Cells were then washed with FBS and resuspended in FBS solution.

Cell analysis in the uterus was performed as described previously [35]. Briefly, uteri were dissected free from the mesometrium and minced into small fragments. Then, the Minced uteri were placed in HBSS containing 200 U/ml hyaluronidase (Sigma Aldrich), 1 mg/ml collagenase type IV (Sigma Aldrich), and 0.2 mg/ml DNase I (Sigma Aldrich) at 37°C for 20 min to digest the uterus tissues. After digestion, cells were washed with PBS supplemented with 0.2% BSA and incubated in the same buffer at 37°C for 15 min before filtration through a 37 μm nylon mesh. After centrifugation, cells were resuspended in PBS supplemented with 0.2% BSA for further staining. Cells suspensions were blocked with anti-mouse CD16/CD32 mAb and then incubated with PerCP-cy5.5-CD45 plus FITC-conjugated anti-CD11c and PE-conjugated anti-F4/80 (eBioscience) mAbs at 4°C for 30 min. Before APC-conjugated anti-CD206 mAb staining, cells were fixed and permeabilized with the Intracelluar Fixation & Permeabilization Buffer set (eBioscience). After staining, cells were rinsed with PBS supplemented with 0.2% BSA and analyzed on a FACS Calibur.

### Total RNA isolation and quantitative PCR (qRT-PCR)

Total RNA isolation and q-PCR were performed as described before [35]. Briefly, total RNAs were extracted with a total RNA extraction kit (BioTeke, Beijing, China) and then reverse transcribed into cDNA with reverse transcription kit (Promega, Madison, WI, USA). According to the manufacturer’s instructions, cDNA was then amplified using SYBR Green MasterMix (ComWin Biotech Co. Ltd, Beijing, China). Q-PCR was performed with a LightCycler 480 (Roche, Indianapolis, IN, USA). The primers used are listed in S1 Table. The mRNA expression of target genes was normalized to glyceraldehyde-3-phosphate dehydrogenase (GAPDH) expression. The fold change was calculated as 2 ^− ΔΔCt^ (cycle threshold).

### Statistical analysis

Variables are expressed as the mean + SD. Statistical analysis was performed using of SPSS (Version 18.0; Chicago, IL, USA). One-way analysis of variance followed by the Mann-Whitney U test was used to analyze macrophage proportion and chemokine expression levels.

## Material and method statement

The authors confirmed that all methods were performed in accordance with the relevant guidelines and regulations.

### Ethics statement

The animal study was reviewed and approved by The Ethics Committee of Suzhou Institute of Systems Medicine (No.: IACUC2102173). Human sample usage was approved by the Ethical Committee of the Human Research Ethics Committee of Dushu Lake Hospital Affiliated to Soochow University & Medical Center of Soochow University (No.: 230071). Written informed consent from the patients was obtained.

## Results

### Macrophage-related genes and signaling pathways change in LPS-induced miscarried mouse uterus

**Fig 1:**
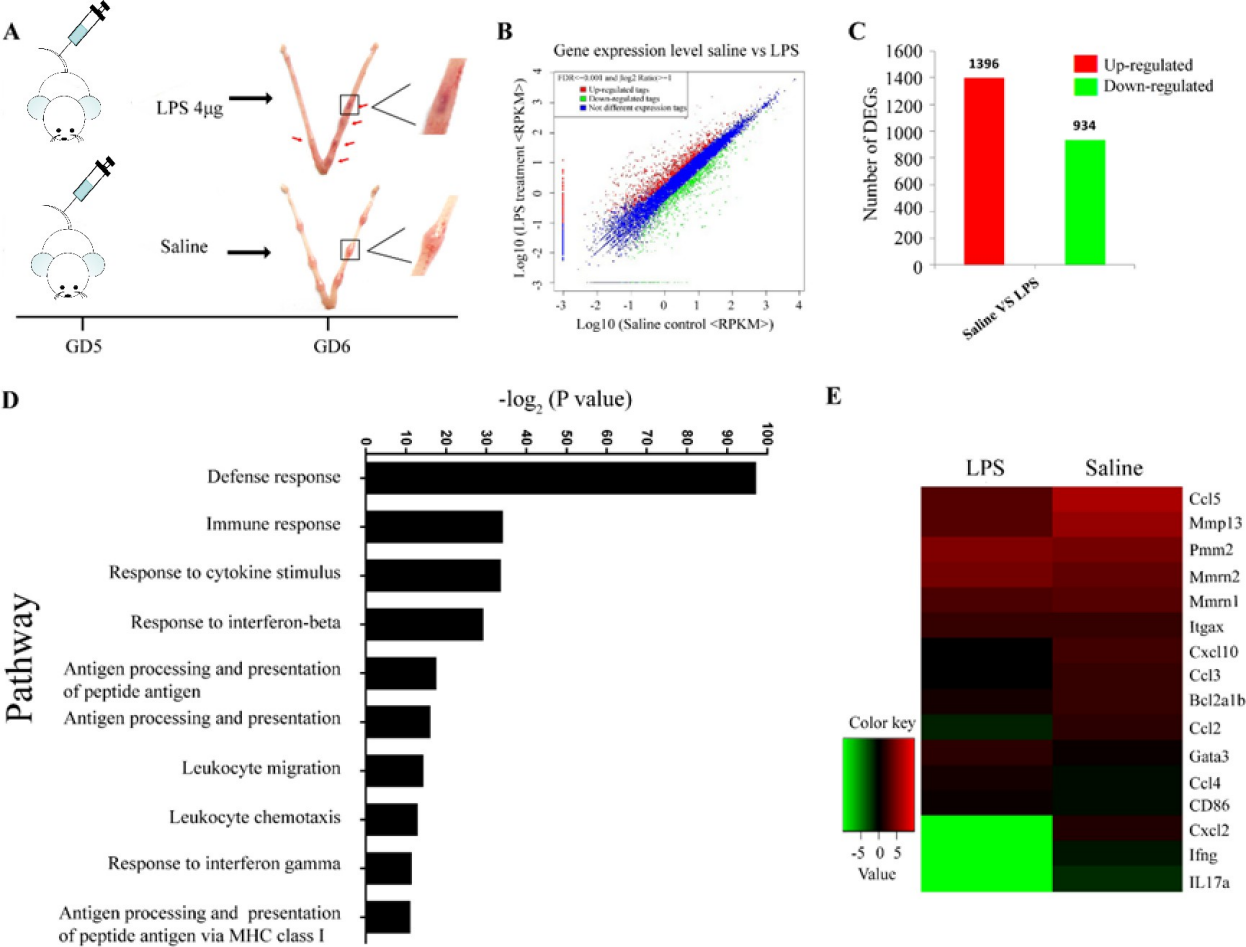
Macrophage-related genes and signaling pathways change in LPS-induced miscarried mouse uterus. (A) The sketch map of the LPS-induced miscarried mice model. (B-E) RNA-seq of LPS-induced miscarried mice uterus and saline control uterus were performed, and the up- and down-regulated genes (B and C), significant changed signal pathway, and genes in LPS-induced miscarried mouse uterus were showed (D and E).

GD5 mice were injected with LPS (4 μg per mouse) or PBS control and sacrificed on GD6. Miscarriage was observed in mice injected with LPS, and nothing abnormal was observed in mice injected with PBS control (Fig 1A and S1 Table). Then transcriptome sequencing was performed with LPS-induced miscarried mice uterus and PBS administration non-miscarried uterus. The sequencing data was analyzed carefully and found that the number of different expression genes was 2330 (Fig 1B and 1C). The expression of many genes associated with monocyte-macrophage polarization and chemotaxis was changed. The novel regulated signaling pathways were defense response, immune response, response to cytokine stimulus, and response to interferon beta (Fig 1D and 1E). These results suggested that the regulation of M1/M2 macrophage polarization and monocyte-macrophage chemotaxis into the uterus may probably be involve in LPS-induced miscarriage.

### The proportion of M1/M2 macrophages changes in LPS-induced miscarried mouse uterus but not in spleen

**Fig 2:**
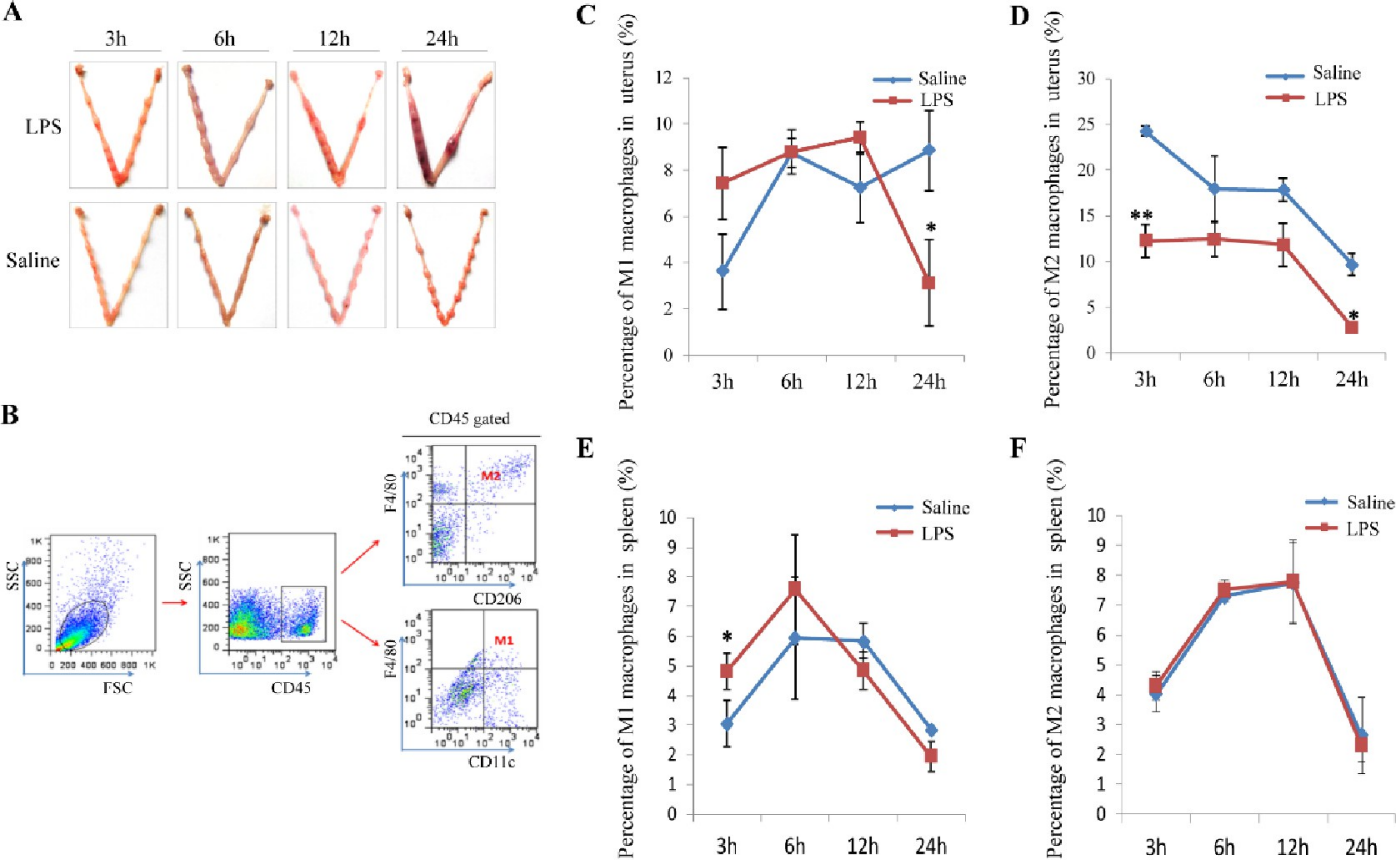
The proportion of M1/M2 macrophages changes in LPS-induced miscarried mouse uterus but not in spleen. (A) The image of the uterus in LPS and saline-administrated mice. (B) The gate strategy of FACS data analysis. (C and D) M1/M2 macrophage percentage in LPS and saline administrated mouse uterus. (E and F) M1/M2 macrophage percentage in LPS and saline administrated mouse spleen. The data (C-F) were shown with mean±SD, Mann-Whitney U test, *P<0.05, **P<0.01.

The transcriptome sequencing results hint that the miscarriage effect of LPS may relate to its functional impact on M1(F4/80^+^CD11c^+^) and M2 (F4/80^+^CD206^+^) macrophages. So, we then analyzed the percentage of M1 and M2 macrophages in CD45^+^ immune cells in the uterus and spleen from pregnant mice injected with LPS or PBS control by FACS experiments (Fig 2A). The percentage of M1 macrophage rose, while the percentage of M2 macrophage declined remarkably with the time of LPS administration in mouse uterus. Both M1 and M2 macrophages had no significant change in the spleen compared to the control group (Figs 2B–2F and S1). The results suggest that LPS may lead to M1/M2 macrophage differentiation imbalance in pregnant mouse uterus. Th1 immune response mediated by M1 macrophages was enhanced while Th2 immune response mediated by M2 macrophages was impaired in LPS-induced miscarried uterus, impacting the immune tolerance of the fetal-maternal interface.

### Monocyte-macrophage migration and expression of related chemokines are up-regulated in LPS-induced miscarried uterus

**Fig 3:**
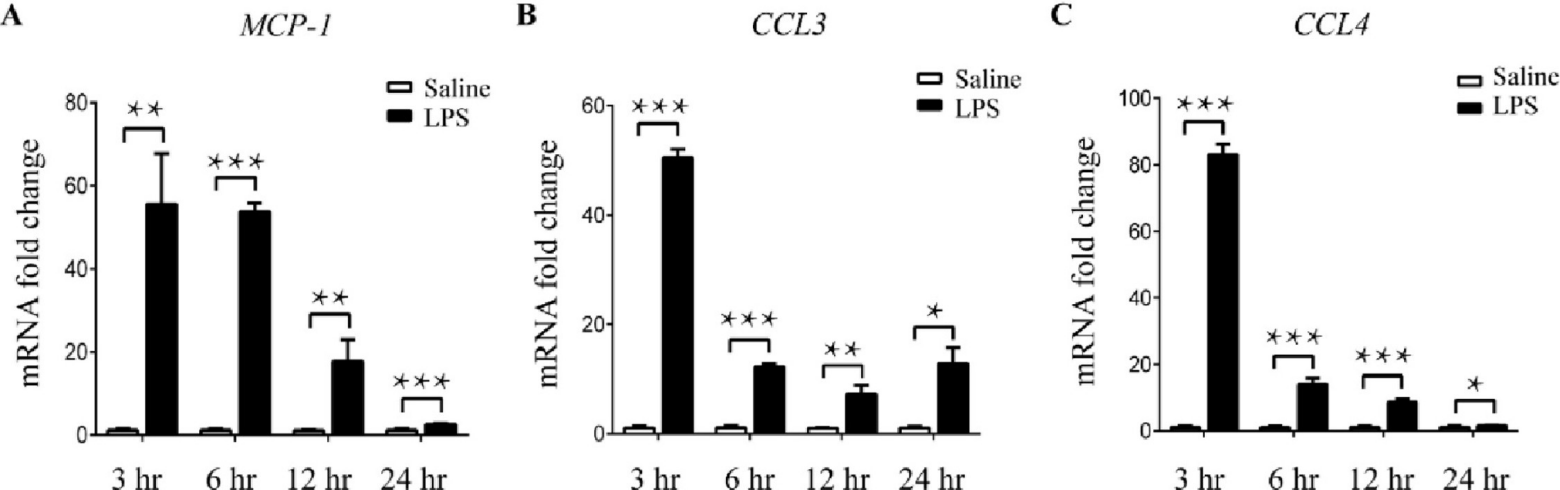
Expression of chemokines associated with monocyte-macrophage migration is up-regulated in LPS-induced miscarried uterus. (A-C) Total RNA of LPS and saline control administrated mouse uterus were extracted, and the expression of Mcp-1, Ccl-3, and Ccl-4 were measured by qRT-PCR assay. The data (A-C) were shown with mean ± SD, Mann-Whitney U test, *P<0.05, **P<0.01, ***P<0.001, ****P<0,0001.

**Fig 4:**
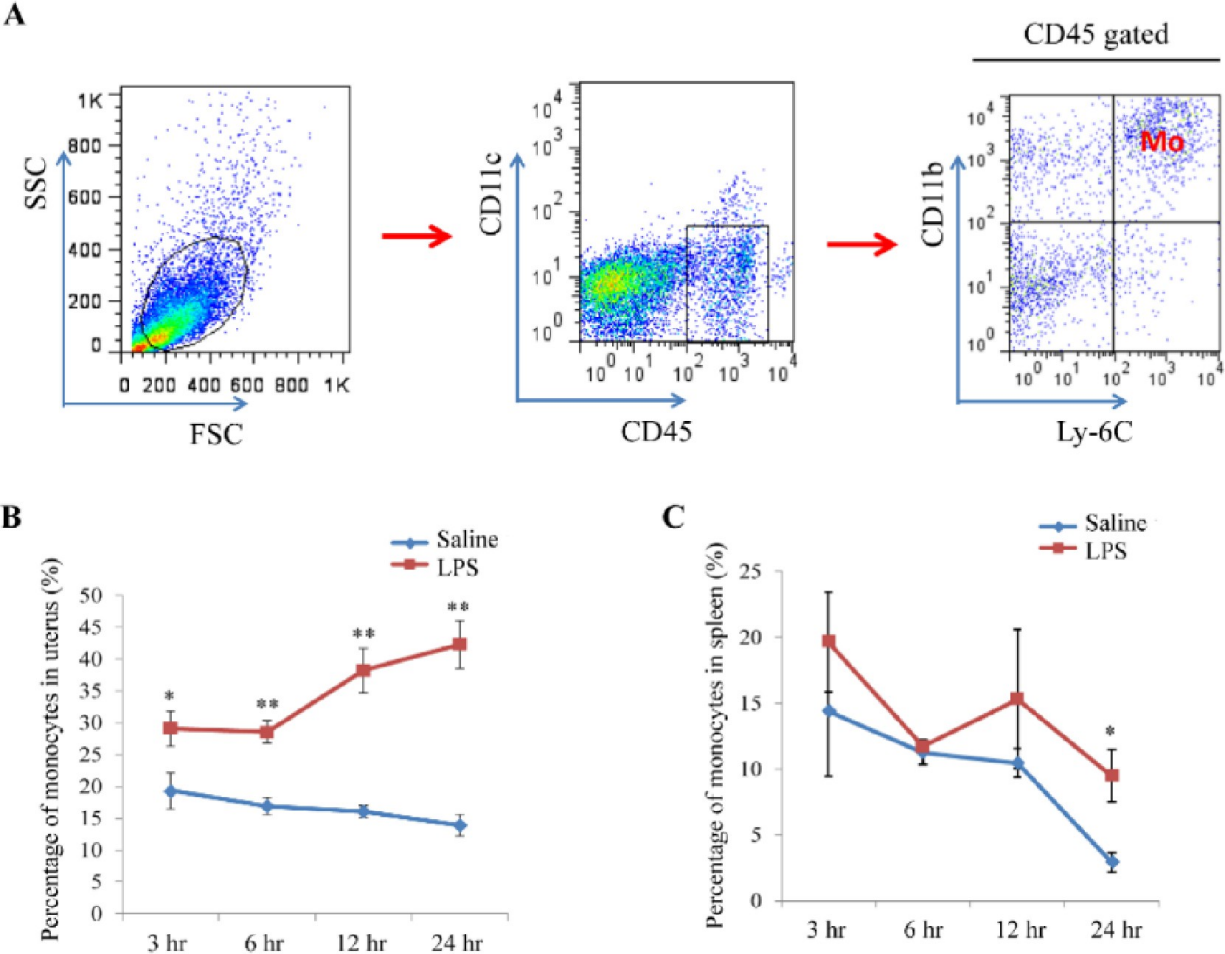
Monocyte-macrophage migration and expression of related chemokines are up-regulated in LPS-induced miscarried uterus. (A) Gate strategy of FACS data analysis. Single-cell suspensions were prepared from the uterus, and the cells were stained with PerCP-cy5.5-CD45, FITC-CD11c, PE-CD11b and APC-Ly-6C on ice for 30 min. Then, the cell suspensions were analyzed with a FACS Calibur, and the gate strategy of data analysis was shown. (B) Monocyte percentages in LPS and saline administrated mouse uterus. (C) Monocyte percentages in LPS and saline administrated mouse spleen. The data (B and C) were shown with mean±SD, Mann-Whitney U test, *P<0.05, **P<0.01.

**Fig 5:**
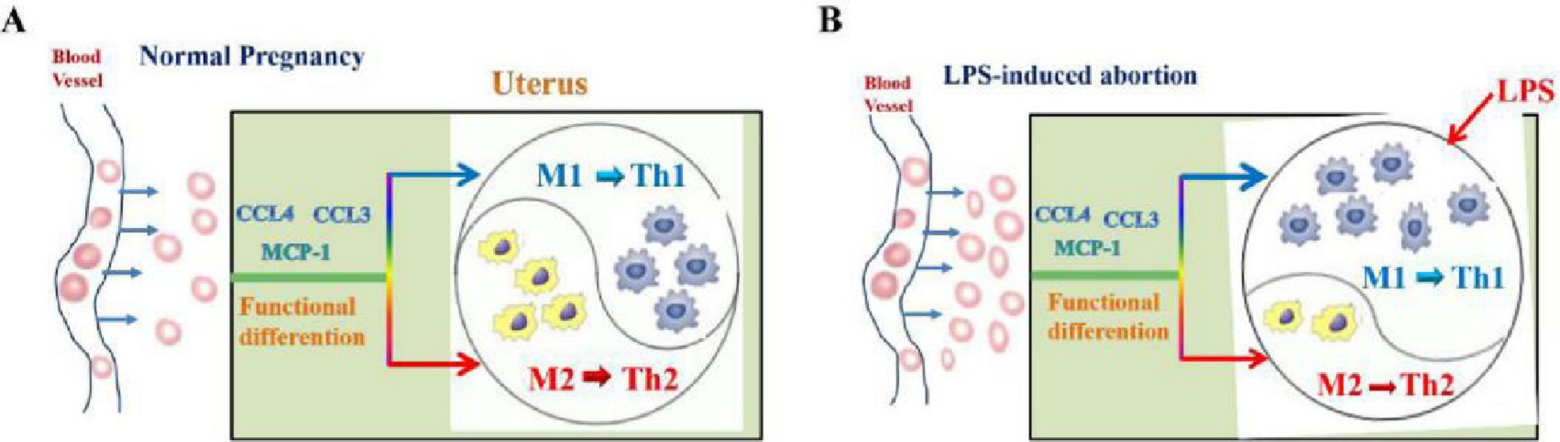
The mechanism sketch of LPS induce-miscarriage. In a normal pregnant uterus, M1/M2 macrophages maintain immune-tolerance balance state (A). In an LPS-induced miscarriage uterus, more M1 macrophages drive a more robust Th1 immune response than Th2, resulting in embryo ejection and miscarriage (B).

Tissue-resident macrophage is thought to have been derived from the migration and differentiation of monocyte into particular tissue. Results above showed that M1/M2 macrophages varied in the uterus of mice injected with LPS, but whether macrophage variation was concerned with its precursors-monocyte was unclear. So, we then measured the expression of monocyte-macrophage chemokine in the uterus from mice injected with LPS or PBS. Expression of chemokine *Mcp-1*, *Ccl3*, and *Ccl4*, which induced monocyte chemotaxis and filtration invasion into the uterus, were remarkably up-regulated in LPS-administrated mouse uterus (Fig 3A–3C and S2 Table). FACS results showed that percentage of monocytes elevated significantly in uterus but had no change in the spleen (Figs 4A–4C and S2). These results suggest that LPS can up-regulate the expression of monocyte-macrophage-associated chemokine and the amount of monocyte migration into the pregnant uterus under the chemotaxis effect. The out-of-balance of M1/M2 percentage in the LPS-administrated uterus may result in miscarriage (Fig 5A and 5B).

### Chemokines associated with monocyte-macrophage migration change in miscarried women’s placentas

**Fig 6:**
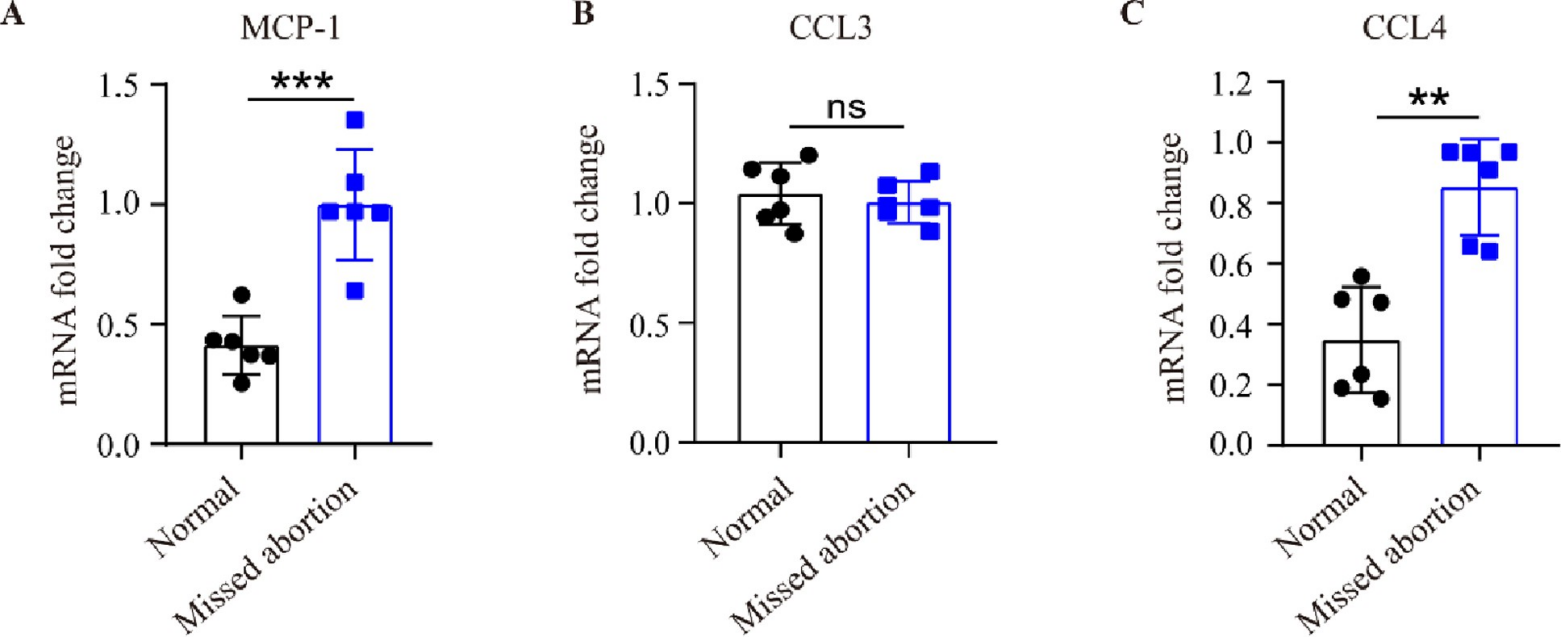
Chemokines associated with monocyte-macrophage migration change in missed miscarried women’s placentas. (A-C) Total RNA of normal and miscarried placentas were extracted, and the expression of *Mcp-1*, *Ccl-3*, and *Ccl-4* were measured by qRT-PCR assay. The data (A-C) were showed with mean±SD, Mann-Whitney U test, ns, not significant, **P<0.01, ***P<0.001.

To confirm that the change of M1/M2 macrophages was associated with miscarriage in humans, we collected 6 normal placentas and 6 miscarried placentas to check the expression of monocyte-macrophage migration-related chemokines by qRT-PCR assay. The results showed that the expression of *Mcp-1* and *Ccl4* was elevated in miscarried placentas when compared with normal placentas (Fig 6A–6C and S3 Table), suggesting the change of monocyte-macrophage migration-related chemokines in human miscarried women’s placentas.

## Discussion

LPS was found to have an adverse effect on pregnancy early in 1943. Many interpretations have been advanced to explain the miscarriage effect of LPS since then, and quite a lot of research has focused on its impact on Th1/Th2 immune balance and fetal-maternal immunotolerance in the uterus. Serum levels of cytokine detection found that serum levels of TNF-α, IFN-γ, IL-4, and NO significantly is elevated in LPS-induced miscarried Balb/c mice when compared to the normal pregnant control group [36]. Meanwhile, serum level of IL-10, a Th2 cytokine, is decreased in LPS-induced miscarried mice. Knockout of the *Il-10* gene increases the miscarriage rate mediated by LPS [37]. CD206^+^ macrophages represent a kind of macrophages that can mediate the Th2 immune response [38]. About 50% of total macrophages were CD206^+^ in early to mid-pregnancy uterus, suggesting that CD206^+^ macrophage may have an crucial protective effect on normal pregnancy [20]. In current study, we examined the proportion change of M1/M2 macrophages in LPS-induced miscarried and normal pregnant uterus and found that LPS up-regulated percentage of M1 macrophage and down-regulated percentage of M2 macrophage in GD5-GD6 mouse uterus, while neither M1 nor M2 macrophage proportions had a significant difference in the spleen. In our research, CD206^+^ macrophage marked drop after LPS administration, and this change may break the M1/M2 macrophages balance in the fetal-maternal interface, causing miscarriage.

Our RT-PCR results showed that the expression of chemokines associated with monocyte-macrophage migration and differentiation was up-regulated by 10–40 times in LPS-injected mouse uterus, especially *Mcp-1*. Expression of *Mcp-1* was up-regulated after LPS administration and MCP-1 induced infiltration of Ly-6C^+^ monocytes into pregnant uterus to differentiate into dendritic cells, neutrophiles, and macrophages [39, 40]. These cells exert respective impact on pregnancy progress and have also been influenced by the micro-environment of pregnant uterus [20]. Some research found that LPS can exert an impact on the differentiation of monocytes to macrophages. Th1 cytokine, like LPS and IFN-ϒ induces monocyte differentiation to M1 macrophage [34, 41, 42]; Th2 cytokine, like IL-10, IL-4, IL-13 can induce monocyte differentiation to M2 macrophage [8, 22, 43, 44].

In this work, we found that percentage of M1 macrophage rose in LPS-induced miscarried uterus when compared to normal pregnant uterus. The percentage of M2 macrophage declined down notably compared to control and dropped continuously with the time after LPS administration. These results suggest that LPS does not promote M1 macrophage generation markedly in pregnant uterus, but LPS did suppress M2 macrophage generation in GD5-GD6 mice uterus. CD206 molecular in macrophage cell surface would reflect the loss of antigen-presenting ability of macrophages and show immune-tolerance character [38]. About half of the macrophages in early to mid-pregnancy uterus are M2 phenotype, M2 macrophages have a potential protective effect on pregnancy. CD14^+^CD206^+^ macrophage in human decidua (dMac) can suppress the killing activity of dNK cells depending on TGF-beta1 signaling pathway. The promoting effect of LPS on M1 macrophage and suppressing effect on M2 macrophage may result in the Th1/Th2 immune imbalance in the pregnant uterus and trigger adverse effects on maternal-fetal health [45, 46]. Also, when human placenta samples were examined, higher expression levels of monocyte-macrophage migration chemokines were observed in miscarried placentas than in normal placentas (Fig 6). These results indicate a relationship between M1/M2 macrophage differentiation and miscarriage.

Our work provides a new line of evidence correlating the aberrant regulation of M1 and M2 macrophages with deleterious miscarriage-inducing effects and will help us in understanding human miscarriage.

## Supporting information

S1 Fig LPS regulates the proportion of M1/M2 macrophages in pregnant mouse uterus but not in spleen. (A-D) M1/M2 macrophage percentage in LPS and saline administrated mouse uterus and spleen were analyzed by FACS.
(DOCX)

S2 Fig Monocytes infiltrated into the uterus are increased in LPS-induced miscarried mouse uterus. (A-B) Monocyte percentage in LPS and saline administrated mouse uterus and spleen were analyzed by FACS.
(DOCX)

S1 Table Information of LPS-induced abortion mice model. (DOCX)

S2 Table Primer sequences for Real-Time qPCR experiments. (DOCX)

S3 Table Clinical information of pregnant women recruited in this study. (DOCX)

## Abrreviations

LPS: Lipopolysaccharides
RNA-seq: RNA sequencing
qRT-PCR: quantitative real time PCR
MCP-1: monocyte chemotactic protein
