# Supplementary material for: Imbalance polarization of M1/M2 macrophages in miscarried uterus
Source: PLoS One. 2024 Jul 25;19(7):e0304590. doi: 10.1371/journal.pone.0304590 (PMC11271943; doi:10.1371/journal.pone.0304590)
Supplement: S1 Fig — (A-D) M1/M2 macrophage percentage in LPS and saline administrated mouse uterus and spleen were analyzed by FACS. (DOCX) [file pone.0304590.s001.docx]

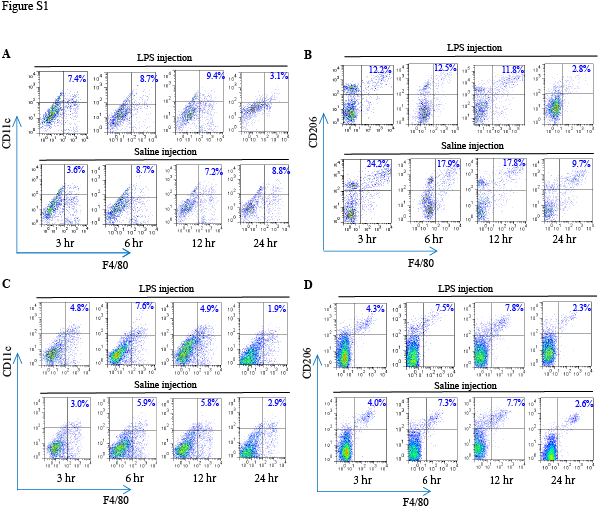


**Figure S1. LPS regulates the proportion of M1/M2 macrophages in pregnant mouse uterus but not in spleen**

(A-D) M1/M2 macrophage percentage in LPS and saline administrated mouse uterus and spleen were analyzed by FACS.
