# Supplementary material for: Imbalance polarization of M1/M2 macrophages in miscarried uterus
Source: PLoS One. 2024 Jul 25;19(7):e0304590. doi: 10.1371/journal.pone.0304590 (PMC11271943; doi:10.1371/journal.pone.0304590)
Supplement: S2 Fig — (A-B) Monocyte percentage in LPS and saline administrated mouse uterus and spleen were analyzed by FACS. (DOCX) [file pone.0304590.s002.docx]

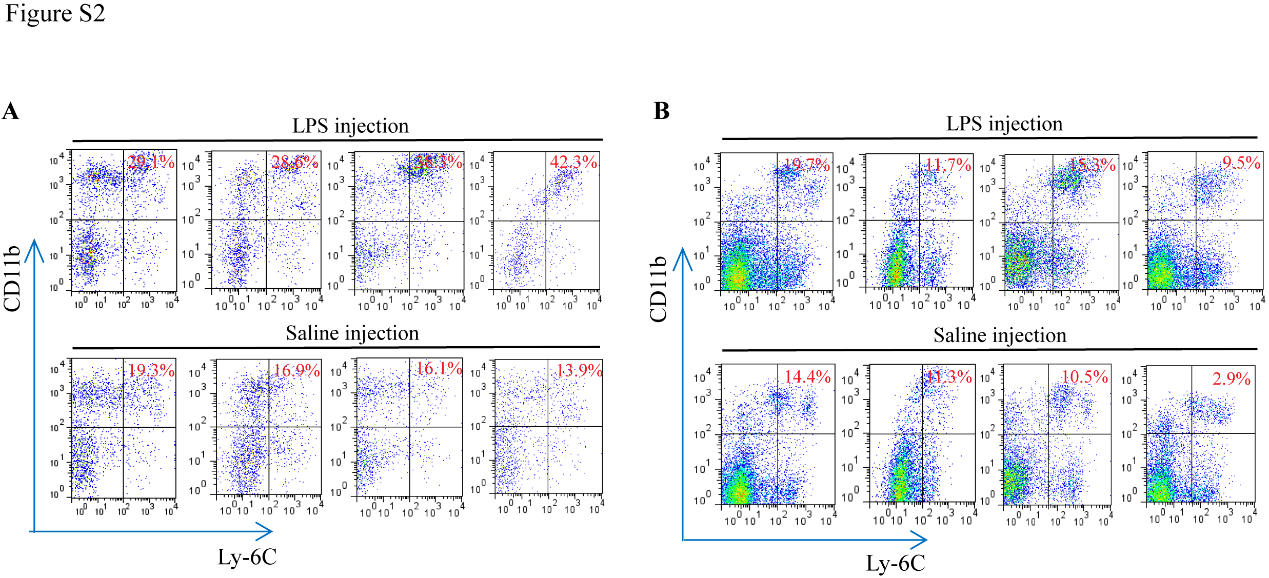


**Figure S2.** **Monocytes infiltrated into the uterus are increased in LPS-induced miscarried mouse uterus**

(A-B) Monocyte percentage in LPS and saline administrated mouse uterus and spleen were analyzed by FACS.
