# Supplementary material for: Imbalance polarization of M1/M2 macrophages in miscarried uterus
Source: PLoS One. 2024 Jul 25;19(7):e0304590. doi: 10.1371/journal.pone.0304590 (PMC11271943; doi:10.1371/journal.pone.0304590)
Supplement: S1 Table — (DOCX) [file pone.0304590.s003.docx]

**Table S1. Information of LPS-induced abortion mice model**

| **Time** | **Treatment** | **Mouse No.** | **Total implantation sites** | **Implantation sites/mouse** |
| --- | --- | --- | --- | --- |
| 3 hr | LPS | 5 | 55 | 11±1.3 |
|  | Saline | 4 | 43 | 10.8±1.7 |
| 6 hr | LPS | 5 | 38 | 7.6±2.1 |
|  | Saline | 4 | 28 | 7±2.7 |
| 12 hr | LPS | 6 | 51 | 8.5±1.6 |
|  | Saline | 5 | 38 | 7.6±1.3 |
| 24 hr | LPS | 5 | 47 | 9.4±1.1 |
|  | Saline | 3 | 26 | 8.7±2.4 |
| Note: LPS group injected with 4μg LPS/ mice, saline group treated with equivalent saline. | | | | |
