# Supplementary material for: Imbalance polarization of M1/M2 macrophages in miscarried uterus
Source: PLoS One. 2024 Jul 25;19(7):e0304590. doi: 10.1371/journal.pone.0304590 (PMC11271943; doi:10.1371/journal.pone.0304590)
Supplement: S2 Table — (DOCX) [file pone.0304590.s004.docx]

**Table S2. Primer sequences for Real-Time qPCR experiments**

| **Gene ID** | **Sequence of primers (5’-3’)** | **Accession No.** |
| --- | --- | --- |
| *mCcl3* | F: TGTACCATGACACTCTGCAAC  R: CAACGATGAATTGGCGTGGAA | NM_011337.2 |
| *mCcl4* | F: TGTACCATGACACTCTGCAAC  R: CAACGATGAATTGGCGTGGAA | NM_013652.2 |
| *mMcp-1* | F: GGTCGCACAGACCTCTCTC  R: TCCTGGGATTTCTCATCAGG | NM_011333 |
| *mGapdh* | F: CCCACTCTTCCACCTTCG  R: CTCTTGCTCAGTGTCCTTG | BC096042 |
| *hCcl3* | F: TCCGTCACCTGCTCAGAAT  R: GCAGCAAGTGATGCAGAGAAC | NM_002983.3 |
| *hCcl4* | F: GCTTCCTCGCAACTTTGTGG  R: TCACTGGGATCAGCACAGAC | NM_002984.4 |
| *hMcp-1* | F: ATCAATGCCCCAGTCACCTG  R: TCTCCTTGGCCACAATGGTC | NM_002982.4 |
| *hGapdh* | F: TGTGGGCATCAATGGATTTGG  R: ACACCATGTATTCCGGGTCAAT | NM_002046.7 |
