# Supplementary material for: Imbalance polarization of M1/M2 macrophages in miscarried uterus
Source: PLoS One. 2024 Jul 25;19(7):e0304590. doi: 10.1371/journal.pone.0304590 (PMC11271943; doi:10.1371/journal.pone.0304590)
Supplement: S3 Table — (DOCX) [file pone.0304590.s005.docx]

**Table S3. Clinical information of pregnant women recruited in this study**

| **Women** | | **Age** | **Gyn history** | **Pregnant weeks** | **Habitual abortion** | **Basic disease** |
| --- | --- | --- | --- | --- | --- | --- |
| Normal | 1# | 30 | 2-0-4-2 | 11+6 | No | No |
|  | 2# | 27 | 1-0-0-1 | 11+4 | No | No |
|  | 3# | 21 | 2-0-0-2 | 9+2 | No | No |
|  | 4# | 22 | 0-0-0-1 | 9+5 | No | No |
|  | 5# | 24 | 2-0-0-1 | 10+1 | No | No |
|  | 6# | 25 | 1-0-0-1 | 10+4 | No | No |
| Miscarried | 1# | 26 | 0-0-0-0 | 13+3 | No | No |
|  | 2# | 27 | 0-0-0-0 | 13+3 | No | No |
|  | 3# | 31 | 1-0-0-1 | 11+2 | No | No |
|  | 4# | 35 | 0-0-2-0 | 10+4 | Yes | No |
|  | 5# | 30 | 0-0-0-0 | 11+6 | No | No |
|  | 6# | 28 | 0-0-0-0 | 11+4 | No | No |
